# Trends and Demographics of Vascular Intestinal Diseases‐Related Mortality Among Adults Living in United States From 1999 to 2020; A CDC Wonder Analysis

**DOI:** 10.1002/jgh3.70267

**Published:** 2025-09-12

**Authors:** Muhammad Shahzad, Kanz Ul Eman Maryam, Ali Hashim, Amna Zaman Khan, Muhammad Abdullah Ali, Ahmed Yar Khan, Muhammad Younas, Syeda Sundus Shah Bokhari, Wania Khalid, Farah Shahzad, Kashmala Zia, Ali Hassan, Muhammad Uzair Khan Niazi, Fahad Rahman, Saad Ahmed Waqas, Raheel Ahmed

**Affiliations:** ^1^ Foundation University Medical College Islamabad Pakistan; ^2^ Al‐Aleem Medical College University of Health Sciences Lahore Pakistan; ^3^ Khyber Medical College Peshawar Pakistan; ^4^ Gomal Medical College, Dera Ismail Khan Pakistan; ^5^ Ayub Medical College Abbottabad Pakistan; ^6^ Department of Anatomy Foundation University Medical College Islamabad Pakistan; ^7^ Fauji Foundation Hospital Rawalpindi Pakistan; ^8^ Dow University of Health Sciences Karachi Pakistan; ^9^ Imperial College London National Heart and Lung Institute London UK

**Keywords:** mortality, trends, vascular intestinal disorders (VID)

## Abstract

**Introduction:**

Vascular intestinal disorders (VID), including mesenteric ischemia, ischemic colitis, and intestinal angiodysplasia, have a global incidence of 8.11/100 000/year and a mortality of 1.26/100 000/year (15.5% death rate), rising from ~1% to ~3% in childhood to ~50% after 95 years. In the US, the incidence of acute vascular insufficiency of the intestine (AVII) is rising, warranting detailed trend analysis.

**Methods:**

CDC WONDER death certificates (1999–2020) for adults > 25 years were analyzed using ICD‐10 code N55. Age‐adjusted mortality rates (AAMRs) per 100 000 were stratified by year, sex, race/ethnicity, and region. Joinpoint Regression (v5.2.0) calculated annual percent changes (APCs); significance was defined as *p* < 0.05.

**Results:**

Overall AAMR declined from 9.35 (1999) to 5.81 (2020). Women had higher AAMRs (7.63; 95% CI: 7.6–7.66) than men (6.5; 95% CI: 6.49–6.56). By race/ethnicity, AAMRs were highest in NH American Indian (7.89; 95% CI: 7.57–8.21), NH Black (7.84; 95% CI: 7.75–7.9), NH White (7.25; 95% CI: 7.22–7.28), Hispanic (5.91; 95% CI: 5.83–6), and NH Asian (3.59; 95% CI: 3.5–3.68). Micropolitan areas had higher AAMRs (7.92) than metropolitan (6.99). Regional AAMRs were highest in the Midwest (7.7; 95% CI: 7.65–7.75), followed by South (7.17; 95% CI: 7.13–7.21), West (7.02; 95% CI: 6.96–7.07), and Northeast (6.85; 95% CI: 6.79–6.9). Kentucky had the highest state AAMR (9.67; 95% CI: 9.43–9.9), Hawaii the lowest (4.59; 95% CI: 4.31–4.87). Oklahoma, Rhode Island, Tennessee, West Virginia, and Wyoming ranked in the top 90th percentile.

**Conclusion:**

Despite an overall decline, VID mortality remains high among women, NH American Indians, rural areas, and the Midwest—underscoring the need for targeted interventions.

## 1 Introduction

Vascular intestinal disorders (VID) include conditions such as mesenteric ischemia, ischemic colitis, and intestinal angiodysplasia, each of which manifests with diverse clinical symptoms [1]. Mesenteric ischemia often causes severe abdominal pain, nausea, vomiting, and, in chronic cases, postprandial pain with weight loss. Ischemic colitis typically manifests as lower abdominal pain and bloody diarrhea, while intestinal angiodysplasia may lead to gastrointestinal bleeding, anemia, and fatigue due to chronic blood loss [2].

The incidence of acute vascular insufficiency of the intestine (AVII) is on the rise in the USA and is associated with significant morbidity and mortality. Seasonal variations have been observed in the onset of several gastrointestinal diseases [3]. The current global incidence and mortality of vascular disorders of the intestine are 8.11 per 100 000 cases/year and 1.26 per 100 000 deaths/year, respectively, translating into a death rate of 15.5%. The death rate increased in parallel with incidence and mortality, from ∼1% to 3% in childhood up to ∼50% after the age of 95 years [4].

According to a study carried out in Scotland, from 2 142 921 deaths over 36 years, 14,530 (0.7%) were due to mesenteric vascular disease with a median (interquartile range) age of 77 and a 2:1 female to male gender ratio [5]. Geographically, the regions of America had the highest burden of VID in young individuals. From 2000 to 2019, there was an increasing prevalence in all areas, with the most pronounced change observed in Southeast Asia [6].

The total prevalence count of VID increased from 20,096 in 1990 to 27,547 in 2021. The total percentage change (TPC) in incidence count rose by 46%, with deaths increasing by 16%. Age‐wise, the 20–54 age group saw a 19% rise in TPC for incidence. For deaths, the 20–54 age group experienced a 31% increase [7].

## 2 Methods

### 2.1 Study Setting and Population

This descriptive study utilized data from death certificates available in the CDC WONDER database [8] to examine trends and demographic patterns in mortality associated with vascular disorders of the intestine among adults in the United States from 1999 to 2020. The analysis was conducted using codes from the International Statistical Classification of Diseases and Related Health Problems, 10th Revision (ICD‐10), specifically N55 [9]. The validity of this ICD code for identifying deaths related to vascular disorders of the intestine has been established in previous epidemiological studies. The data, obtained from death certificates of US residents, included information on causes of death and demographic details. Cases were identified using the Multiple Cause‐of‐Death Public Use Records, where vascular intestinal disorders were documented as either the primary or a contributing cause of death. Since this study relied on a publicly available, de‐identified dataset, Institutional Review Board approval was not required. Furthermore, the study follows the STROBE (Strengthening the Reporting of Observational Studies in Epidemiology) guidelines for reporting [10].

### 2.2 Data Extraction

The data were categorized based on age, sex, race, urbanization level, and geographic region. Racial and ethnic groups included non‐Hispanic (NH) White, NH Black or African American, Hispanic or Latino, NH American Indian or Alaskan Native, NH Asian or Pacific Islander, and NH individuals identifying with more than one race. Age was divided into 7 groups: 25–34, 35–44, 45–54, 55–64, 65–74 and 85+. All findings were derived from death certificates, a data source that has been utilized in previous research using the CDC WONDER dataset.

### 2.3 Statistical Analysis

To examine national trends and demographic variations in mortality related to vascular disorders of the intestine, crude mortality rates and age‐adjusted mortality rates (AAMRs) per 100 000 individuals were analyzed from 1999 to 2020. These rates were stratified by age, sex, race, urbanization level, and geographic region, with corresponding 95% confidence intervals (CIs). Crude mortality rates were calculated annually by dividing the number of VID‐related deaths by the respective US population for that year.

AAMRs were derived using a weighted average of age‐specific mortality rates, standardized to the US population of 2000. To assess trends, the Joinpoint Regression Program (Version 5.2.0) was used to estimate annual percentage changes (APCs) and average annual percentage changes (AAPCs) in AAMRs, along with their 95% CIs [11]. A statistically significant increase or decrease in APCs was determined if the corresponding regression slope significantly differed from zero, based on two‐tailed *t*‐tests, with a significance level set at *p* < 0.05.

## 3 Result

### 3.1 Overall Trends

**FIGURE 1:**
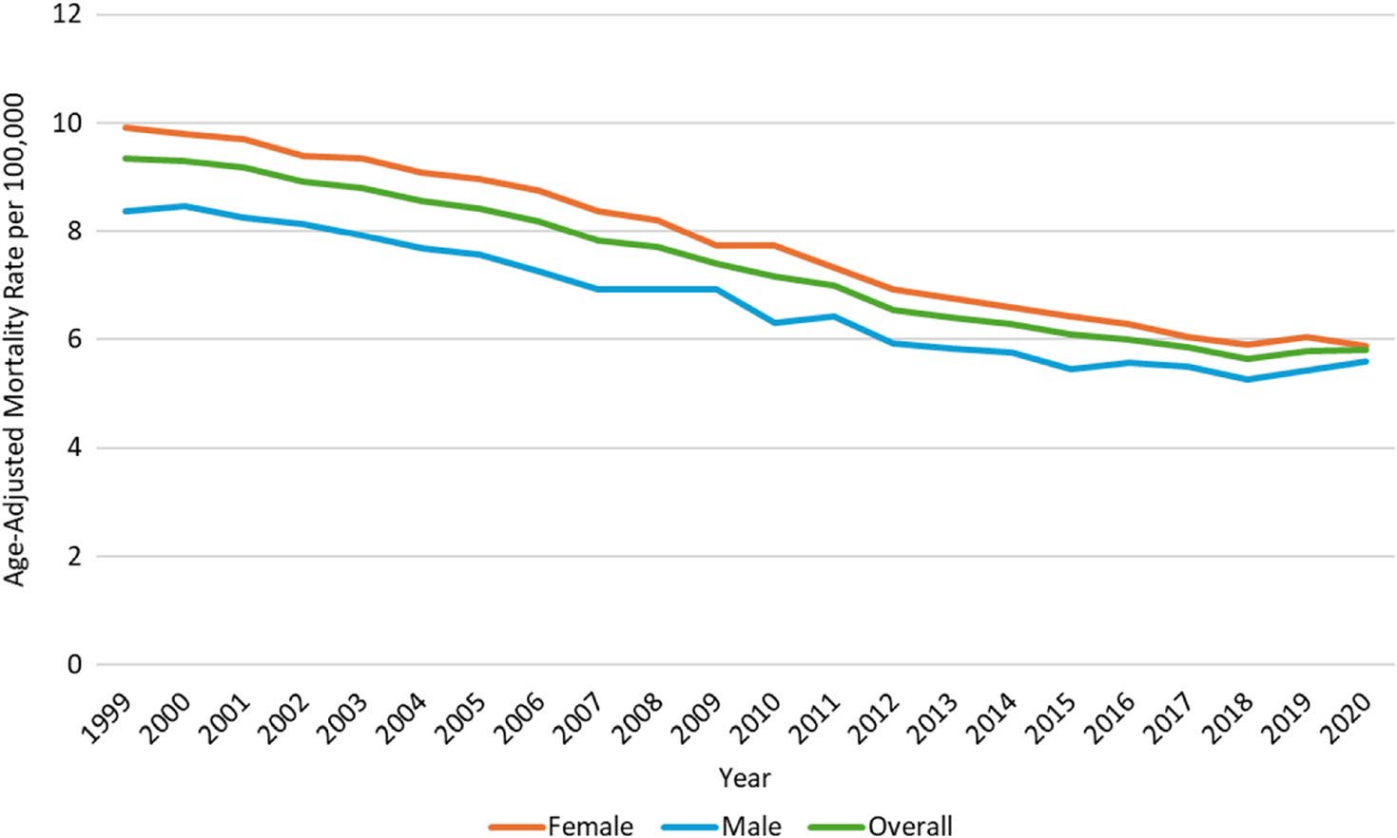
Overall and sex‐stratified vascular intestinal diseases‐related AAMRs per 100 000 in adults in the United States, 1999–2020.

In 1999, the overall AAMR was 9.35, which decreased to 5.81 in 2020. The overall AAMR decreased from 1999 to 2005 (APC: −1.78; 95% CI: −2.26 to −1.30), followed by a sharp decline till 2013 (APC: −3.40; 95% CI: −3.77 to −3.03). Again, a decline was seen till 2018 (APC; −2.37; 95% CI: −3.32 to −1.42). However, a non‐significant incline was observed in AAMR till 2020 (APC: 1.18; 95% CI: −2.01 to 4.48) (Figure 1, Table S1 and Figure S1).

### 3.2 Gender‐Specific Trends

Women had consistently higher AAMRs than men throughout the study period (overall AAMR women: 7.63; 95% CI: 7.6 to 7.66, men: 6.5; 95% CI: 6.49 to 6.56). The AAMR for women decreased from 1999 to 2005 (APC: −1.73; 95% CI: −2.33 to −1.12) followed by a further decline till 2017 (APC: −3.32; 95% CI: −3.57 to −3.06). A final nonsignificant decline in AAMR followed until 2020 (APC: −0.34; 95% CI: −2.18 to 1.54). Likewise, the AAMR for men decreased from 1999 to 2018 (APC: −2.65; 95% CI: −2.85 to −2.45), followed by a non‐significant increase till 2020 (APC: 3.52; 95% CI: −3.23 to 10.74) (Figure 1, Table S1 and Figure S2).

### 3.3 Race/Ethnicity‐Specific Trends

When stratified by race/ethnicity, the AAMR was highest among NH American Indians, followed by NH Black, NH White, Hispanic, and lastly NH Asian population. (Overall AAMR NH American Indian/Alaska Natives: 7.89, 95% CI: 7.57–8.21; NH Asian/Pacific Islander: 3.59, 95% CI: 3.5–3.68, NH Black/African American: 7.84, 95% CI: 7.75 to 7.92, NH White: 7.25, 95% CI: 7.22–7.28, Hispanic/Latinos: 5.91, 95% CI: 5.83–6).

*Non‐Hispanic American Indian*: The AAMR for NH American Indian was 8.18 in 1999, which decreased to 6.09 in 2020 (APC: −2.13; 95% CI: −2.81 to −1.45).

*Hispanic:* The AAMR for Hispanics was 8.66 in 1999, which sharply decreased to 4.78 in 2017 (APC: −3.45; 95% CI: −3.7 to −3.02); however, it showed a non‐significant increase to 5.09 in 2020 (APC: 2.47; 95% CI: −3.15 to 8.44).

*Non‐Hispanic Black/African American*: The AAMR for NH Black/African American was 9.06 in 1999, which decreased to 5.27 in 2016 (APC: −3.19; 95% CI: −3.40 to −2.98). The AAMR showed a non‐significant increase to 5.75 in 2020 (APC: 0.88; 95% CI: −1.01 to 2.83).

*Non‐Hispanic Asian/Pacific Islander*: The AAMR for NH Asian was 3.38 in 1999, which decreased to 2.7 in 2010 (APC: −1.77; 95% CI: −2.73 to −0.80). However, a notable decrease was observed to 2.09 in 2013 (APC: −9.46; 95% CI: −20.54 to 3.17). The AAMR then remained stable to 2020 (APC: −0.38; 95% CI: −1.92 to 1.18).

**FIGURE 2:**
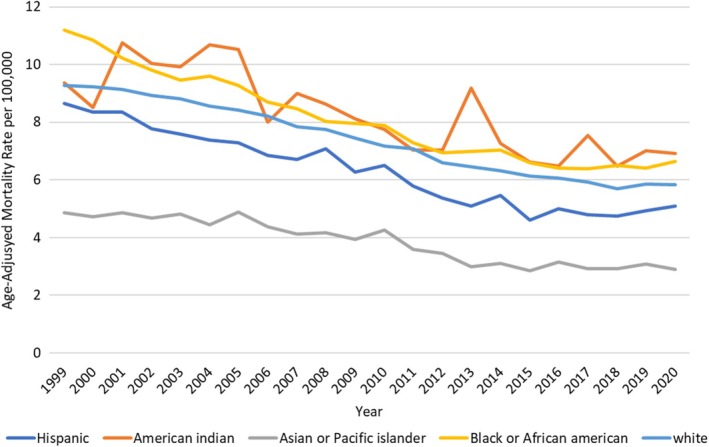
Vascular intestinal diseases‐related AAMRs per 100 000 stratified by race in adults in the United States, 1999–2020.

*Non‐Hispanic White*: The AAMR decreased from 6.87 in 1999 to 6.13 in 2005 (APC: −1.64; 95% CI: −2.19 to −1.09), which further sharply decreased to 4.87 in 2013 (APC: −3.30; 95% CI: −3.74 to −2.85). Similarly, the AAMR decreased to 4.41 in 2018 (APC: −2.35; 95% CI: −3.44 to −1.25). However, an increase was observed in AAMR to 4.68 in 2020 (APC: 0.93; 95% CI: −2.40 to 4.39) (Figure 2, Table S1 and Figure S3).

### 3.4 Urban/Rural Trends

Upon stratification, AAMR was highest among Micropolitan (nonmetro), followed by Noncore (nonmetro), small metro, medium metro, large central metro, and large fringe metro.

*Large Central metro*: The AAMR decreased from 9.81 in 1999 to 5.12 in 2018 (APC: −3.51; 95% CI: −3.70 to −3.33), which showed a non‐significant increase to 5.25 in 2020 (APC: 1.59; 95% CI: −5.34 to 9.03).

*Medium Metro*: The AAMR decreased from 9.69 in 1999 to 6.08 in 2018 (APC: −2.60; 95% CI: −2.77 to −2.43); however, the AAMR showed a non‐significant increase to 6.2 in 2020 (APC: 1.34; 95% CI: −4.84 to 7.93).

*NonCore (Nonmetro)*: The AAMR was 9.39 in 1999, which declined to 6.71 in 2018 (APC: −1.63; 95% CI: −1.96 to −1.31). The AAMR further showed a non‐significant increase to 7.88 in 2020 (APC: 5.07; 95% CI: −6.08 to 17.55).

*Large Fringe Metro*: The AAMR decreased from 8.72 in 1999 to 8.1 in 2004 (APC: −1.42; 95% CI: −2.75 to −0.07). The AAMR further decreased steeply to 5.44 in 2014 (APC: −3.87; 95% CI: −4.42 to −3.32). Lastly, it decreased to 4.88 in 2020 (APC: −1.91; 95% CI: −3.00 to −0.82).

*Small Metro*: The AAMR showed a non‐significant increase from 8.98 in 1999 to 9.53 in 2001 (APC: 3.47; 95% CI: −2.07 to 9.34), which further decreased to 6.53 in 2017 (APC: −2.64; 95% CI: −2.87 to −2.41). Lastly, it remained stable till 2020 (APC: 0.68; 95% CI: −2.04 to 3.49).

**FIGURE 3:**
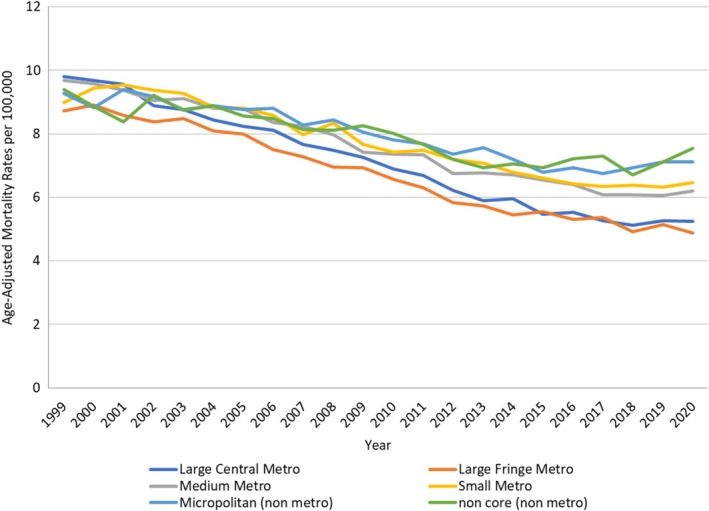
Vascular intestinal diseases‐related AAMRs per 100 000 stratified by urban/rural status in adults in the United States, 1999–2020.

*Micropolitan (Nonmetro)*: The AAMR decreased from 9.27 in 1999 to 8.8 in 2006 (APC: −0.93; 95% CI: −1.79 to −0.06). The AAMR sharply decreased to 6.74 in 2017 (APC: −2.31; 95% CI: −2.82 to −1.79). However, it showed a non‐significant increase to 7.12 in 2020 (APC: 2.37; 95% CI: −0.93 to 5.80) (Figure 3, Table S1 and Figure S4).

### 3.5 Geographical/Census Region‐Specific Trends

Throughout the study, the highest AAMR was noted in the Midwestern region at 7.7 (95% CI: 7.65–7.75), followed by the Southern Region at 7.17 (95% CI: 7.13–7.21), the Western region at 7.02 (95% CI: 6.96–7.07), and the Northeastern region at 6.85 (95% CI: 6.79–6.9).

**FIGURE 4:**
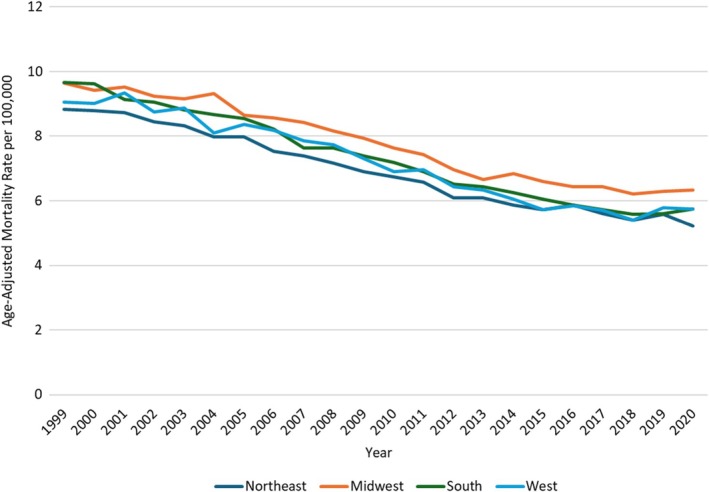
Vascular intestinal diseases‐related AAMRs per 100 000 stratified by geographical trends in adults in the United States, 1999–2020.

In the Midwestern region, AAMR showed a non‐significant decrease from 1999 to 2004 (APC: −0.92; 95% CI: −2.01 to 0.18), followed by a sharp decrease till 2015 (APC: −3.06; 95% CI: −3.47 to −2.66) and then AAMR remained statistically stable till 2020 (APC: −0.74; 95% CI: −1.95 to 0.48). In the Southern region, the AAMR decreased from 1999 to 2005 (APC: −2.40; 95% CI: −3.00 to −1.80), followed by a further decrease till 2018 (APC: −3.21; 95% CI: −3.42 to −2.99), and finally showed a non‐significant increase till 2020 (APC: 2.21; 95% CI: −1.71 to 6.30). In the Western region, AAMR declined from 1999 to 2007 (APC: −1.90; 95% CI: −2.78 to −1.01), with a further decrease till 2015 (APC: −3.96; 95% CI: −5.01 to −2.88). AAMR then remained statistically stable till 2020 (APC: −0.38; 95% CI: −2.19 to 1.45). In the Northeastern region, AAMR decreased from 1999 to 2003 (APC: −1.54; 95% CI: −3.10 to 0.06), followed by a sharp decrease till 2014 (APC: −3.13; 95% CI: −3.54 to −2.72), and then finally a further decrease till 2020 (APC: −1.67; 95% CI: −2.60 to −0.73) (Figure 4, Table S1 and Figure S5).

### 3.6 States‐Specific Trends

**FIGURE 5:**
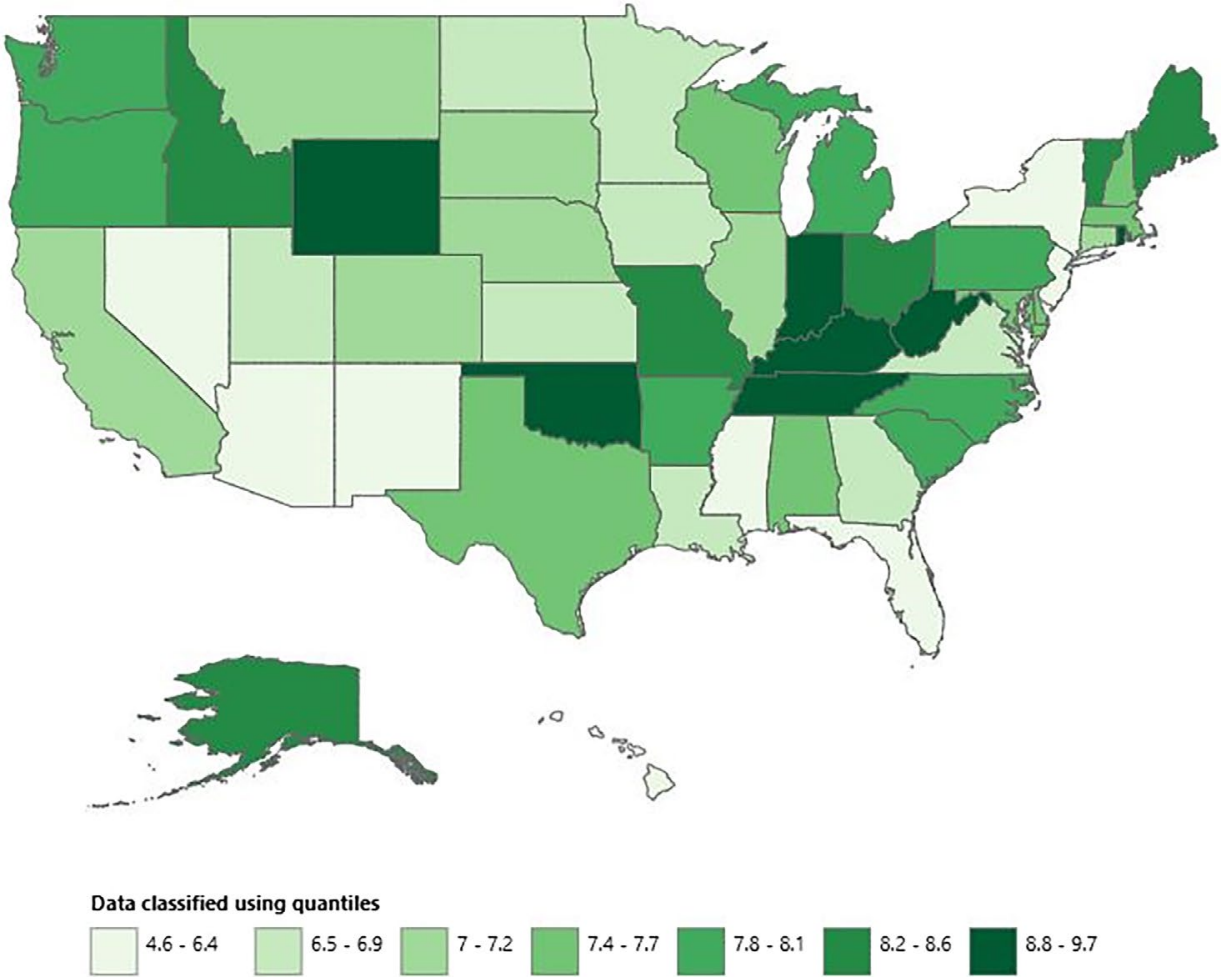
Vascular intestinal diseases‐related AAMRs per 100 000 stratified by states in adults in the United States, 1999–2020.

The highest AAMR was reported by Kentucky at 9.67 (95% CI: 9.43 to 9.9) and the lowest AAMR was reported by Hawaii at 4.59 (95% CI: 4.31 to 4.87). States like Oklahoma, Rhode Island, Tennessee, West Virginia, and Wyoming fell into the top 90th percentile of the AAMRs. Whereas, Arizona, Nevada, New Jersey, and New York had their AAMRs in the lower 10th percentile (Figure 5, central illustration).

## 4 Discussion

This nationwide study evaluated the age‐adjusted mortality rates (AAMR) among the US population for vascular pathologies of the intestine. A general decline in mortality was observed in the study from 1999 to 2018, followed by a slight increase till 2020. The decline in AAMR can be attributed to technological developments in diagnostic fields, prompt surgical interventions, and better management of vascular conditions [12]. The development of revascularization techniques significantly reduced the fatality rate due to acute and chronic mesenteric ischemia in this region during the early 2000s [13]. These findings also reflect improvement in public health and the management of risk factors for vascular diseases. From 2018 onwards, a slight increase in AAMR was observed in the population. This increase can be linked to the COVID‐19 pandemic, which significantly affected the treatment of non‐Covid conditions. Some studies also suggest an association of COVID‐19 with vascular thrombosis [14].

Sex‐specific trends reveal that females had higher AAMR as compared to males throughout the study period. Females of reproductive age are at higher risk of vascular thromboembolic disease due to hormonal contraceptives. Females outlive males and are more prone to develop chronic vascular conditions, especially in the postmenopausal period. Hormone replacement therapies in the postmenopausal period also increase the risk of ischemic diseases [15]. The higher mortality rates in females may reflect gender‐based differences in access to healthcare facilities in the United States [16].

Significant racial and ethnic inequities were observed throughout the study. The Non‐Hispanic American Indian population had the highest AAMRs, reflecting broader socioeconomic factors such as limited access to care and economic disparities. This may be attributed to the paucity of access to sub‐specialty healthcare by virtue of distance and transportation barriers [17]. To overcome the systemic barriers to health equity, targeted interventions, policies, and efforts are required. Increased oxidative stress and chronic inflammation, leading to vascular stiffness and aging, are posited to be the result of social, economic, and environmental disadvantages faced by the NH Black American patients. This can be the reason for the higher AAMRs among this population [18]. The fluctuations in mortality among Hispanics, particularly the sharp decline in the years 2010 to 2013, can be due to the increasing success of highly efficacious endovascular treatments of vascular intestinal pathologies, such as percutaneous angioplasty and stenting [19].

Geographic trends demonstrated the highest AAMR in the Midwestern region of the US (7.7) followed by the Southern region, and was relatively lower in other regions. The Midwestern and Southern regions of the US have the highest population residing in rural areas. As compared to urban settlements, individuals in rural areas have a lack of access to healthcare facilities and limited public health awareness [20]. This can contribute to delayed medical interventions, ultimately increasing the risk of mortality. A decline in the mortality rate was observed in these regions from 2004 to 2015 (APC −3.06), possibly due to improvements in healthcare facilities.

A striking disparity was observed between urban and rural populations, with rural areas such as micropolitan and non‐Core (non‐metro) regions exhibiting the highest AAMRs and Large Central Metro and Large Fringe Metro showing the lowest AAMRs. This can be explained by the disparity in healthcare utilization between urban and rural status, the shortage of gastroenterologists in rural compared to urban areas, inadequacy of insurance coverage, and longer travel distances affecting the time of initiation of therapy [21]. States such as Kentucky displayed the highest AAMR, which can be explained by the low‐resourced Appalachian counties that are dense in vulnerable populations at the risk of intestinal pathologies [22]. Hawaiians, on the other hand, had the lowest AAMRs among states, which is attributable to their dietary habits, leading to a lower incidence of vascular pathologies [23].

## 5 Limitations

Although this study provides a meaningful perspective on mortality trends related to vascular pathologies of the intestine, there are a few limitations. Firstly, it depends on death certificate data, which may under‐report or misclassify vascular intestinal diseases. Additionally, geographical trends may be altered since the database provides the state in which the individual died, leading fatalities to be incorrectly categorized from the state in which the individual resided Secondly, the study did not take into consideration risk factors like hyperlipidemia, smoking, alcohol consumption, obesity, and confounding conditions like diabetes, cardiovascular diseases, and hypertension, which would have helped further elaborate on the underlying causes of observed trends. The death statistics from 2019 onwards may have been impacted by the COVID‐19 pandemic, possibly influencing or altering mortality trends.

## 6 Conclusion

The findings of this study underscore the fluctuating mortality trends posed by vascular intestinal disorders in the US Notably, the increasing mortality rates, particularly in the Midwest, rural areas, women, and the non‐Hispanic American Indian populations, highlight the need for targeted public health efforts. Although medical advancements have led to declines in mortality during some periods, the rising prevalence of obesity, alcohol consumption, and other social determinants of health has contributed to posing a threat. Addressing these trends requires focused efforts on prevention, targeted interventions, and treatment, particularly for high‐risk populations.

## Conflicts of Interest

The authors declare no conflicts of interest.

## Supporting information


**Data S1:** Supporting Information.

## Data Availability

Data sharing is not applicable to this article as no new data were created or analyzed in this study.
