# Supplementary material for: Trends and Demographics of Vascular Intestinal Diseases‐Related Mortality Among Adults Living in United States From 1999 to 2020; A CDC Wonder Analysis
Source: JGH Open. 2025 Sep 12;9(9):e70267. doi: 10.1002/jgh3.70267 (PMC12426610; doi:10.1002/jgh3.70267)
Supplement: Supplementary file 1 — Data S1: Supporting Information. [file JGH3-9-e70267-s001.docx]

**Table 1: Annual Percentage Changes (APCs) in Vascular Intestinal Disease-Related Mortality Trends in United States 1999-2020**

| VARIABLE | YEAR INTERVAL | APC (95%) | p-value |
| --- | --- | --- | --- |
| Overall  3 Joinpoints | 1999-2005 | -1.7814* | **0.000006** |
|  | 2005-2013 | -3.4019* | **< 0.000001** |
|  | 2013-2018 | -2.3795* | **0.000199** |
|  | 2018-2020 | 1.1849 | 0.436667 |
| Female  2 Joinpoints | 1999-2005 | -1.7343* | **0.000029** |
|  | 2005-2017 | -3.3186* | **< 0.000001** |
|  | 2017-2020 | -0.3401 | 0.701535 |
| Male  1 Joinpoint | 1999-2018 | -2.6502* | **< 0.000001** |
|  | 2018-2020 | 3.5185 | 0.294245 |
| Hispanic or Latino  1 Joinpoint | 1999-2017 | -3.4515* | **< 0.000001** |
|  | 2017-2020 | 2.4774 | 0.374167 |
| American Indian or Alaska Native  0 Joinpoints | 1999-2020 | -2.1391* | **0.000003** |
| Asian or Pacific Islander  2 Joinpoints | 1999-2010 | -1.7759* | **0.001576** |
|  | 2010-2013 | -9.4614 | 0.125022 |
|  | 2013-2020 | -0.3800 | 0.609230 |
| Black or African American  1 Joinpoint | 1999-2016 | -3.1939* | **< 0.000001** |
|  | 2016-2020 | 0.8885 | 0.341522 |
| White  3 Joinpoints | 1999-2005 | -1.6445* | **0.000042** |
|  | 2005-2013 | -3.3027* | **< 0.000001** |
|  | 2013-2018 | -2.3569* | **0.000660** |
|  | 2018-2020 | 0.9394 | 0.553776 |
| Northeastern Region  2 Joinpoints | 1999-2003 | -1.5351 | 0.057890 |
|  | 2003-2014 | -3.1273* | **< 0.000001** |
|  | 2014-2020 | -1.6714* | **0.001949** |
| Midwestern Region  2 Joinpoints | 1999-2004 | -0.9220 | 0.093913 |
|  | 2004-2015 | -3.0630* | **< 0.000001** |
|  | 2015-2020 | -0.7388 | 0.215023 |
| Southern Region  2 Joinpoints | 1999-2005 | -2.4034* | **0.000001** |
|  | 2005-2018 | -3.2063* | **< 0.000001** |
|  | 2018-2020 | 2.2108 | 0.251528 |
| Western Region  2 Joinpoints | 1999-2007 | -1.8978* | **0.000458** |
|  | 2007-2015 | -3.9563* | **0.000002** |
|  | 2015-2020 | -0.3816 | 0.661222 |
| Large Central Metro  1 Joinpoint | 1999-2018 | -3.5163* | **< 0.000001** |
|  | 2018-2020 | 1.5934 | 0.643134 |
| Large Fringe Metro  2 Joinpoints | 1999-2004 | -1.4275* | **0.039499** |
|  | 2004-2014 | -3.8779* | **< 0.000001** |
|  | 2014-2020 | -1.9195* | **0.002147** |
| Medium Metro  1 Joinpoint | 1999-2018 | -2.6057* | **< 0.000001** |
|  | 2018-2020 | 1.3454 | 0.660128 |
| Small Metro  2 Joinpoints | 1999-2001 | 3.4782 | 0.204835 |
|  | 2001-2017 | -2.6435* | **< 0.000001** |
|  | 2017-2020 | 0.6895 | 0.600303 |
| Micropolitan (Nonmetro)  2 Joinpoints | 1999-2006 | -0.9314* | **0.037810** |
|  | 2006-2017 | -2.3116* | **< 0.000001** |
|  | 2017-2020 | 2.3794 | 0.147182 |
| NonCore (Nonmetro)  1 Joinpoint | 1999-2018 | -1.6395* | **< 0.000001** |
|  | 2018-2020 | 5.0705 | 0.365516 |

*Statistically significant p-values are highlighted bold

APC, annual percent change; CI, confidence interval

**Table 2: Age-Adjusted Mortality Rates (AAMR) in Vascular Intestinal Disease-Related Mortality Trends in the United States 1999-2020**

| State | Age Adjusted Mortality Rate |
| --- | --- |
| Alabama | 7.5 |
| Alaska | 8.4 |
| Arizona | 6.1 |
| Arkansas | 8 |
| California | 7.1 |
| Colorado | 7.2 |
| Connecticut | 7 |
| Delaware | 7.7 |
| District of Columbia | 8.2 |
| Florida | 5.4 |
| Georgia | 6.5 |
| Hawaii | 4.6 |
| Idaho | 8.2 |
| Illinois | 7 |
| Indiana | 8.8 |
| Iowa | 6.9 |
| Kansas | 6.5 |
| Kentucky | 9.7 |
| Louisiana | 6.7 |
| Maine | 8.6 |
| Maryland | 7.6 |
| Massachusetts | 7.5 |
| Michigan | 8.1 |
| Minnesota | 6.5 |
| Mississippi | 6.3 |
| Missouri | 8.4 |
| Montana | 7 |
| Nebraska | 7.2 |
| Nevada | 5.6 |
| New Hampshire | 7.6 |
| New Jersey | 5.9 |
| New Mexico | 6.4 |
| New York | 5.8 |
| North Carolina | 8 |
| North Dakota | 6.8 |
| Ohio | 8.5 |
| Oklahoma | 9.1 |
| Oregon | 7.8 |
| Pennsylvania | 7.9 |
| Rhode Island | 9.2 |
| South Carolina | 7.9 |
| South Dakota | 7.1 |
| Tennessee | 8.9 |
| Texas | 7.4 |
| Utah | 6.9 |
| Vermont | 8.3 |
| Virginia | 6.6 |
| Washington | 7.8 |
| West Virginia | 9.2 |
| Wisconsin | 7.5 |
| Wyoming | 8.9 |
